# From Transcriptional Reprogramming to Fat Quality Improvement: Dietary *Artemisia ordosica* Krasch. Optimizes Fatty Acid Profile in Cashmere Goats

**DOI:** 10.3390/ani16071097

**Published:** 2026-04-02

**Authors:** Lianguang Jiang, Yanli Zhao, Qingyue Zhang, Shangxiong Zhang, Xiaoyu Guo, Yongmei Guo, Sumei Yan

**Affiliations:** 1 Key Laboratory of Animal Nutrition and Feed Science at Universities of Inner Mongolia Autonomous Region, College of Animal Science, Inner Mongolia Agricultural University, Hohhot 010018, China; 13403443375@163.com (L.J.);; 2 National Technology Innovation Center for Prataculture, Hohhot 010018, China

**Keywords:** *Artemisia ordosica* Krasch., cashmere goat, subcutaneous adipose tissue, n3-polyunsaturated fatty acid

## Abstract

This experiment investigated the effects of dietary *Artemisia ordosica* Krasch. (AOK) supplementation on the n3-polyunsaturated fatty acid (n3-PUFA) profile of subcutaneous adipose tissue (SADT) in Arbas cashmere goats and explored the underlying transcriptional mechanisms. Forty healthy, weaned kids (120 ± 10 days of age; similar body weight) were randomly allocated to two groups (*n* = 20): a control group (CON, basal diet) and an AOK group (AOK, basal diet with 3% of the roughage replaced by AOK). The feeding trial spanned 104 days, consisting of a 14-day adaptation period and 90 days of data acquisition. Compared with the CON group, AOK significantly reduced the content of saturated fatty acids (SFAs) and n6-polyunsaturated fatty acids (n6-PUFAs)/n3-PUFAs (n6/n3). In contrast, the levels of n3-PUFAs in the SADT of cashmere goats increased markedly (*p* < 0.05). Compared with the CON group, AOK exhibited significantly higher activities of hormone-sensitive lipase (HSL) (*p* = 0.027), adenylyl cyclase 2 (ADCY2) (*p* = 0.010), adenylyl cyclase 5 (ADCY5) (*p* = 0.046), cluster of differentiation 36 (CD36) (*p* = 0.013), solute carrier family 27 member 4 (SLC27A4) (*p* = 0.021), and fatty acid binding protein 4 (FABP4) (*p* = 0.040), along with significantly lower activities of fatty acid synthase (FAS) (*p* = 0.002), lipoprotein lipase (LPL) (*p* = 0.048), and stearoyl-coa desaturase (SCD) (*p* = 0.026) in SADT. Compared with the CON group, the activities of superoxide dismutase (SOD) (*p* = 0.032), catalase (CAT) (*p* = 0.010), glutathione peroxidase (GSH-PX) (*p* = 0.029), and total antioxidant capacity (T-AOC) (*p* = 0.002) were significantly increased in the AOK group. Transcriptomic profiling revealed that AOK supplementation downregulated mRNA levels of *ADCY2*, *ADCY*5, *LPL*, *FAS*, *SCD*, *stearoyl-CoA desaturase* 1 (*SCD*1), stearoyl-*CoA desaturase* 2 (*SCD*2), *glycogen synthase* 1 (*GYS*1), *acyl-CoA oxidase* 1 (*ACOX*1), *acetyl-CoA carboxylase* (*ACC*), *diacylglycerol acyltransferase* 1 (*DGAT*1), *fatty acid desaturase* 1 (*FADS*1), *solute carrier family* 27 *member* 2 (*SLC*27*A*2), *erythroblastic leukemia viral oncogene homolog* 4 (*ERBB*4), and *carnitine palmitoyltransferase* 1*B* (*CPT*1*B*) (*p* < 0.05). It also markedly induced *acyl-CoA synthetase long-chain family member* 4 (*ACSL*4) (*p* < 0.01) in SADT. Genes significantly enriched in the adenosine-monophosphate-activated protein kinase (AMPK) signaling pathway included *LPL*, *SCD*1, *CPT*1*B*, and *GYS*1 (*p* = 0.010). Genes significantly enriched in the phosphatidylinositol 3-kinase-akt (PI3K-Akt) signaling pathway included *GYS*1 and *ERBB*4 (*p* = 0.015). *CPT*1*B*, *ADCY*2, and *GYS*1 were identified as the genes significantly enriched in the insulin resistance signaling pathway (*p* = 0.048). *LPL* was the only gene significantly enriched in the cholesterol metabolism pathway (*p* = 0.049). Genes showing a tendency toward significant enrichment in the peroxisome-proliferator-activated receptor (PPAR) signaling pathway included *ACSL*4, *CPT*1*B*, *SCD*1, and *LPL* (*p* = 0.051). These interconnected cascades improve insulin sensitivity, stimulate triglyceride (TG) hydrolysis, and modulate n3-PUFA levels. Supplementation with AOK enhances n3-PUFA content by accelerating TG breakdown while simultaneously restraining FA oxidation in SADT. Consequently, AOK supplementation can be effectively used to enhance the nutritional value of cashmere goat meat through improved n3-PUFA deposition in SADT.

## Simple Summary

N3-polyunsaturated fatty acids (n3-PUFAs) contribute to the regulation and prevention of cardiovascular diseases in humans by lowering lipid levels, exerting anti-inflammatory effects, and inhibiting thrombosis. Since they cannot be synthesized endogenously, n3-PUFAs must be obtained from dietary sources, with meat consumption serving as an effective means of intake. The Inner Mongolia Arbas white cashmere goat is highly valued for the tenderness and high digestibility of its meat. Subcutaneous adipose tissue (SADT) constitutes an essential part of cashmere goat meat. However, intensive feedlot finishing often compromises eating quality and flavor, primarily by reducing n3-PUFA content in adipose tissue and thereby diminishing the meat’s characteristic nutritional profile. As the fatty acid (FA) profile of fat largely determines meat quality, strategies for increasing the n3-PUFA level in SADT have become a key research focus in this field. *Artemisia ordosica* Krasch. (AOK), a drought-tolerant shrub widely distributed in northern China, exhibits strong antioxidant activity that can inhibit lipid peroxidation and raise tissue FA levels. Supplementation with AOK extract has already improved n3-PUFA status in goats, yet its effects on SADT and the underlying mechanism remain unexplored. Therefore, this study aims to quantify how dietary AOK alters the FA profile of SADT, and to elucidate its underlying mechanisms through transcriptomic analysis, thereby providing a theoretical foundation for the use of AOK as a natural feed additive to enhance product quality during cashmere goat production.

## 1. Introduction

N3-polyunsaturated fatty acids (n3-PUFAs) are essential for regulating and preventing human diseases, including inflammatory and neurological disorders such as asthma and glomerulonephritis [1]. However, since these fatty acids (FAs) cannot be synthesized de novo by humans, dietary intake remains the primary means of obtaining them, and the consumption of n3-PUFA-rich meat represents a feasible nutritional strategy. The Inner Mongolia cashmere goat, a versatile breed valued nationwide for its tender and digestible meat, serves as a potential source of such lipids. Subcutaneous adipose tissue (SADT), a major component of goat carcasses, significantly influences meat flavor and nutritional quality [2].

However, under intensive farming systems, the current industry standard, n3-PUFA levels in SADT have been progressively declining [1]. This trend is concerning because n3-PUFA profiles in SADT are key determinants of sensory attributes, carcass traits, muscle yield, and overall nutritional value [3,4]. Consequently, understanding the regulatory mechanisms underlying n3-PUFA deposition in the SADT of Inner Mongolia cashmere goats is essential for developing nutritional or genetic strategies to sustain optimal n3-PUFA levels and ensure the production of high-quality cashmere goat meat.

*Artemisia ordosica* Krasch. (AOK), a semi-shrub of the Asteraceae family, grows abundantly on the sandy steppes of northern and north-western China. The plant is rich in alcohols, phenolics, organic acids, saccharides, and terpenoids that exhibit potent antioxidant activity and suppress lipid peroxidation, thereby preserving polyunsaturated fatty acids (PUFAs) [5,6]. Supplementation with AOK extract increased the arteriovenous difference of C18:1t9 and C18:1c9 across the bovine mammary gland, providing more monounsaturated precursors for downstream n3-PUFA synthesis [7] and elevated n3-PUFA levels in goat tissues [8]. Zhang et al. (2018) [9] also found that AOK could enhance the activity of total antioxidant capacity (T-AOC), catalase (CAT), and glutathione peroxidase (GSH-Px) in cashmere goat blood, while reducing malondialdehyde (MDA) levels.

Furthermore, experimental evidence indicates that flaxseed significantly increases n3-PUFA content in sheep tissues [10]. Additionally, research has shown that sea buckthorn pulp not only elevates n3-PUFA levels in broiler chicken pectoral muscles [11] but also optimizes the fatty acid profile in pig plasma [12]. However, research on the effects of AOK on the FA composition and antioxidant capacity of SADT, as well as the underlying mechanisms, remains limited. Kadegowda et al. [13] discovered that inhibiting stearoyl-CoA desaturase1 (SCD1) activity reduces the activity of genes involved in FA synthesis while elevating the expression of FA oxidation-related genes. Elevated fatty acid desaturase (FADS) enzyme activity promotes the conversion of C18:1n9 to C20:5n3 and C22:6n3 during PUFA synthesis [14]. Wang [15] demonstrated that elevated elongase of very-long-chain fatty acids 2 (ELOVL2) activity in cashmere goats’ adipose tissue accelerates the conversion of C18:1n-9 to n3-PUFAs. Huang [16] further demonstrated that inhibition of fatty acid synthase (FAS) activity can decrease de novo lipogenesis, and adjustment of the adenosine-monophosphate-activated protein kinase (AMPK) signaling pathway downregulates key lipogenic regulators such as sterol regulatory element-binding protein 1 (SREBP1), acetyl-CoA carboxylase (ACC), and FAS, which in turn enhances insulin sensitivity and attenuates lipid accumulation. Conversely, upregulation of *carnitine palmitoyltransferase* 1*B* (*CPT*1*B*) enhances lipolysis, ultimately increasing FA content. The redox processes in adipose tissue not only influence FA levels by modulating *CPT*1*B* and *cluster of differentiation* 36 (*CD*36) expression [17] but also regulate inflammatory and insulin signaling pathways [18]. Despite these prospects, research on the application of AOK in ruminants remains limited. The effects of AOK on the FA composition and antioxidant capacity of SADT, along with the underlying mechanisms, remain poorly understood.

This study was conducted using cashmere goats to investigate the impacts of dietary AOK supplementation on the FA composition of SADT and to explore its underlying mechanisms through transcriptomic analysis. The objective was to provide a theoretical foundation for utilizing AOK resources in cashmere goat production. Based on evidence that bioactive plant compounds can regulate lipid metabolism, it was hypothesized that AOK influences enzyme activities and gene expression associated with n3-PUFA synthesis and antioxidant function. Accordingly, this experiment was designed to assess how dietary AOK modifies SADT fatty acid profiles and to elucidate the transcriptional pathways involved, thus providing insights into the potential application of AOK as a natural feed additive in cashmere goat production systems.

## 2. Materials and Methods

The AOK used in this research was sourced from an experimental sandy base in Ordos, Inner Mongolia, China, and the experiment was conducted at the experimental farm of Inner Mongolia Agricultural University (Hohhot, China). The study procedure was approved by the Animal Ethics and Welfare Committee of Inner Mongolia Agricultural University (approval number: IMAU-2019-032).

### 2.1. Experimental Design, Dietary Formulation, and Feeding Regimen

This study employed a single-factor completely randomized design. All experimental animals were 120-day-old goats with an average body weight of 18.6 ± 0.1 kg and were randomly assigned to 2 groups with 4 replicates per group (5 goats per replicate). The control group (CON) was fed a basal diet, while the AOK group received a basal diet with 3% crude feed replaced by AOK. The dose of AOK was based on the results of a preliminary experiment [19]. According to the feeding standards for meat sheep and goats (NY/T 816, 2021 [20]), the dietary nutrient levels met the requirements of growing goats. Following a 14-day acclimation period, the 90-day treatment period was divided into three fattening phases: early (d 1–30), middle (d 31–60), and late (d 61–90). The ingredients and chemical composition of the basal diet are presented in Table 1, while the FA composition of the experimental diet and AOK is presented in Table A1. The goats were housed individually in animal rooms and fed ad libitum. The diet was provided twice daily at 08:00 and 15:00.

### 2.2. Sample Collection

Upon completion of the experimental trial, 8 goats from each group (2 randomly selected from each replicate) were humanely slaughtered by exsanguination. All experimental goats were fasted for 24 h and deprived of water for 2 h prior to slaughter. Then, 1 h after slaughter, about 80 g of SADT was taken and wrapped in tin foil and stored at −20 °C for the determination of FA content. Additionally, about 10 g of subcutaneous fat was collected and stored at −80 °C for analysis of lipid metabolism enzyme activity, antioxidant indices, and transcriptome profiling.

### 2.3. Calculation of Fatty Acid Intake

According to the dry matter intake (DMI) of cashmere goats, the FA intake was calculated using formula (1). The DMI of cashmere goats was determined based on the findings of a previous experiment [21].

FA intake (g/W^0.75^) = (DMI × dietary FA content × individual FA composition)/weight^0.75^ (1)

### 2.4. Chemical Analysis

#### 2.4.1. Determination of FA Content in SADT

Following cryogenic grinding with liquid nitrogen, 0.5 g of homogenized SADT powder was collected. FA methyl esters (FAMEs) were prepared from the SADT powder according to the methodology of Yao et al. [22]. The procedure was as follows: The SADT powder sample was weighed and transferred into a hydrolysis tube. Subsequently, 0.7 mL of 10 N KOH and 5.3 mL of methanol solution were added. The mixture was heated in a water bath (55 °C, 1.5 h) and then cooled to room temperature. Next, 0.58 mL of 24 N sulfuric acid solution was added and mixed thoroughly. The mixture was again heated in a 50 °C water bath for 1.5 h, then cooled to room temperature. Finally, 3 mL of hexane was added, and the mixture was vortexed for 5 min and centrifuged at 1500 rpm for 6 min. The n-hexane phase was transferred, filtered through a membrane filter, and subjected to instrumental analysis.

Using the external standard quantitative analysis method, FAMEs were determined. Chromatographic separation was performed on an SPTM-2560 capillary column (100 m × 0.25 mm × 0.2 μm, Supelco, Bellefonte, PA, USA). High-purity nitrogen was used as the carrier gas at a constant flow rate of 1.2 mL/min. For the flame ionization detector (FID), the auxiliary gas parameters were set as follows: air flow rate at 450 mL/min, make-up gas (nitrogen) flow rate at 25 mL/min, and hydrogen flow rate at 40 mL/min. The injector temperature was set at 250 °C with a split ratio of 30:1, and the detector temperature was also maintained at 250 °C. Each sample was analyzed by injecting a 1 μL aliquot. FAME mixed standard solutions used for calibration and quantification were acquired from Sigma-Aldrich. The oven temperature was programmed as follows: an initial temperature of 150 °C for 5 min, then increased to 175 °C at a rate of 2 °C/min and held for 15 min, followed by a rise to 200 °C at 6 °C/min for 20 min. Finally, the temperature was elevated to 220 °C at 5 °C/minutes and maintained for 10 min. FAs in the samples were identified and quantified by comparing their retention times with those of FA standards.

The determination of FA included 30 types of individual FA components (as shown in Table A1). In addition, the contents of saturated fatty acids (SFAs), unsaturated fatty acids (UFAs), monounsaturated fatty acids (MUFAs), PUFAs, n6-polyunsaturated fatty acids (n6-PUFAs), and n3-PUFAs were calculated. Furthermore, the ratios of n6-PUFAs/n3-PUFAs (n6/n3), UFAs/SFAs (U/S), and PUFAs/SFAs (P/S) were also calculated.

#### 2.4.2. Determination of Enzyme Activity Related to Lipid Metabolism and Antioxidase Activity in SADT

Commercial ELISA kits (Ruixin Biological Technology Co., Ltd., Quanzhou, China) were used to determine the activities of stearoyl coA desaturase (SCD), ACC, FAS, ELOVL2, elongation of a very-long-chain FA protein 5 (ELOVL5), lipoprotein lipase (LPL), hormone-sensitive lipase (HSL), malate dehydrogenase (MDH), CD36, long-chain FA transporter 4 (SLC27A4), and FA binding protein (FABP4), in strict accordance with the manufacturer’s instructions. The activities of antioxidant-related enzymes, including GSH-Px, CAT, total superoxide dismutase (T-SOD), and T-AOC, as well as the content of MDA, were measured using commercial assay kits according to the manufacturer’s protocols. Specifically, GSH-Px activity was determined by the dithionitrobenzoic acid method, CAT activity by the visible light method, T-SOD activity by the hydroxylamine method, T-AOC by the colorimetric method, and MDA content by the thiobarbituric acid method. The kits were provided by Nanjing Jiancheng Bioengineering Institute (Nanjing, Jiangsu, China), and the instruments used were fully functional microplate readers (EL-X800, Bio-Tek, Winooski, VT, USA).

#### 2.4.3. Transcriptome Sequencing and Analysis

Approximately 1 g of subcutaneous adipose tissue was weighed, and total RNA was extracted using the Promega Eastep Super Total RNA Extraction Kit (Promega, Madison, WI, USA) according to the manufacturer’s instructions. Genomic DNA was removed using the DNase I kit (Promega, Madison, WI, USA). RNA concentration and purity were measured using ND-2000 (NanoDrop Technologies, Wilmington, DE, USA). The RIN value was determined using the 2100 Bioanalyser (Agilent, Santa Clara, CA, USA).

RNA samples with OD 260/280 ≥ 1.8, OD 260/230 ≥ 1.0, RIN ≥ 6.5, 28S:18S ≥ 1.0, total amount ≥ 1 μg, and concentration ≥ 35 ng/uL were selected for library construction. RNA-seq transcription libraries were prepared from 1 μg of total RNA using the TruSeqTM RNA Sample Preparation Kit (Illumina, San Diego, CA, USA). Target fragments of cDNA with sizes ranging from 200 to 300 bp on 2% agarose gels were selected and then PCR-amplified using Phusion DNA polymerase (NEB). After quantification using TBS380, paired-end RNA-seq sequencing libraries were sequenced on the Illumina HiSeq Xten (2 × 150 bp) (Illumina). The sequencing work was completed by Majorbio Bio-Pharm Technology Co., Ltd. (Shanghai, China). The raw transcriptome paired-end sequencing reads were trimmed and quality-controlled using SeqPrep (Version 1.2) and Sickle (Version 1.33) with default parameters. Clean reads were subsequently aligned to the goat reference genome in a stranded mode using HISAT software (Version 2.1.0) to obtain mapped reads. For each sample, the mapped reads were subjected to sequencing library quality assessments, including insert fragment length inspection and randomness tests, using StringTie (Version 2.1.2).

Differential gene expression analysis between the two groups (CON vs. AOK) was performed using the DESeq2 R package (Version 1.10.1). Genes with *p* ≤ 0.05 and |log2 (fold change)| > 1 identified by DESeq were designated as differentially expressed genes. KEGG pathway enrichment analysis was conducted by comparing with the entire transcriptome database using Goatools (Version 0.6.5) and KOBAS (Version 2.1.1). The KEGG metabolic pathway was considered significantly enriched when *p* ≤ 0.05, tendentially significantly enriched when 0.05 < *p* ≤ 0.10, and not significantly enriched when *p* > 0.10.

Genes such as *CPT1B*, *SCD1*, and *LPL* in SADT were selected to validate the transcriptome results. Three reference genes, *β-actin*, *YWHAZ*, and B2M, were used for real-time quantitative fluorescent analysis alongside the target genes. The primer sequences of reference genes and target genes were cited from Wang et al. [12] (see Table A2). The relative expression levels of target gene mRNA were expressed as 2^−∆∆CT^ using the relative comparative threshold cycle (CT) method as previously described [23]. The qRT-PCR data were normalized using the geometric mean CT values of the three reference genes [24].

### 2.5. Statistical Analysis

Each individual animal was defined as the experimental unit. The experimental data were initially organized using Excel and subjected to T-test analysis using the statistical program of SAS 9.2 software. All determinations were performed in triplicate to ensure repeatability, and the mean values were used for statistical analysis. Statistical results show that *p* ≤ 0.05 indicates significant differences between groups, *p* > 0.10 indicates insignificant differences between groups, and 0.05 < *p* < 0.10 indicates significant differences between groups.

## 3. Results

### 3.1. Intake of FA

The FA intake of cashmere goats is presented in Table 2. No significant differences (*p* > 0.05) were observed between the two groups in the intake of individual FA, including SFAs, UFAs, MUFAs, PUFAs, n3-PUFAs, and n6-PUFAs. Similarly, there were no significant differences in the n-6/n-3, U/S, and P/S ratios (*p* > 0.05).

### 3.2. Effect of AOK on FA Composition in SADT of Cashmere Goats

The effect of dietary supplementation with AOK on the FA composition of SADT in cashmere goats is shown in Table 3. Compared with the CON group, the content of C14:0, C16:0, C18:0, and C21:0 significantly decreased in the AOK group (*p* < 0.05), whereas the contents of other SFAs were significantly higher than those in the CON group (*p* < 0.05). For MUFAs, AOK supplementation significantly increased the contents of C14:1, C15:1, C16:1, C17:1, C20:1 and C24:1 (*p* < 0.05), but did not influence the contents of C18:1n9t (*p* = 0.237) or C18:1n9c (*p* = 0.654). For PUFAs, although the content of C20:4n6 tended to be higher in the AOK group (*p* = 0.063), all other PUFAs were significantly increased compared with the CON group (*p* < 0.05). Compared with the CON group, the SFAs in the AOK group were significantly reduced (*p* = 0.008), while UFAs, MUFAs, PUFAs, n3-PUFAs, and n6-PUFAs were significantly increased (*p* < 0.05). In terms of ratios, n6/n3 (*p* = 0.002) was significantly decreased, whereas U/S (*p* = 0.002) and P/S (*p* < 0.001) were significantly increased.

### 3.3. Effect of AOK on the Activity of Enzymes Related to Lipid Metabolism and Anti-Oxidase Activity in SADT of Cashmere Goats

**Figure 1:**
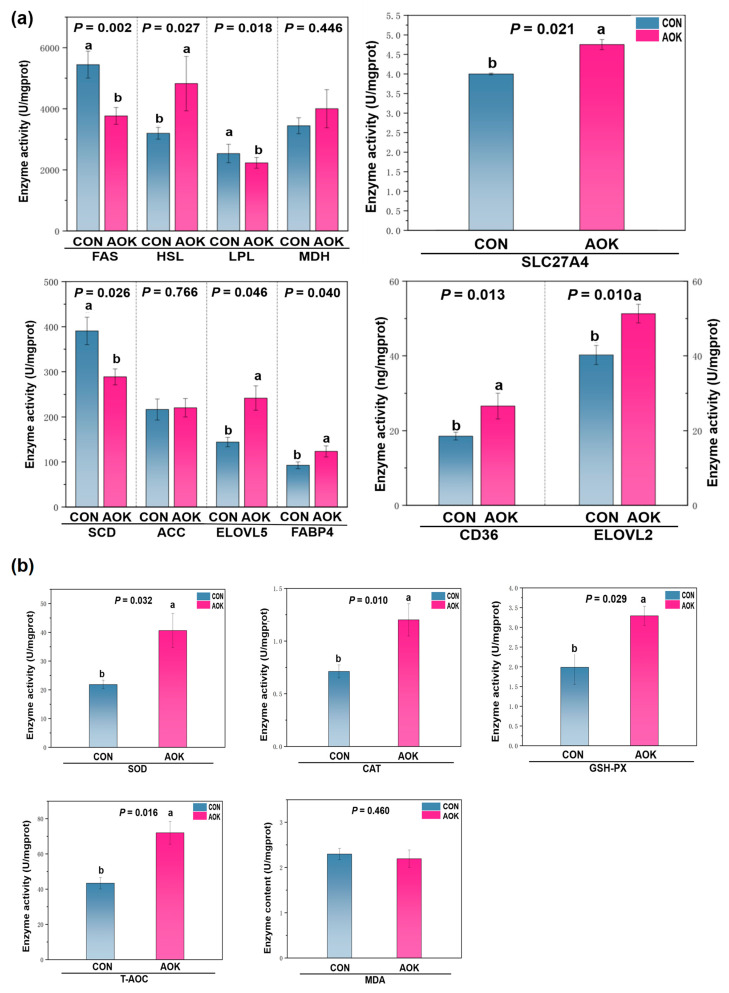
Effect of AOK on the activity of enzymes related to lipid metabolism and anti-oxidase activity in SADT of cashmere goats. (**a**) Effect of AOK on the activities of enzymes related to lipid metabolism in SADT of cashmere goats. (**b**) Effect of AOK on the activity of antioxidant-related enzymes in SADT of cashmere goats. FAS = fatty acid synthase; ACC = acetyl-CoA carboxylase; LPL = lipoprotein lipase; HSL = hormone-sensitive lipase; SCD = stearoyl-CoA desaturase; ELOVL2 = elongase of very-long-chain fatty acids 2; ELOVL5 = elongase of very-long-chain fatty acids 5; MDH = malate dehydrogenase; CD36 = cluster of differentiation 36; SLC27A4 = solute carrier family 27 member 4; FABP4 = fatty acid binding protein 4; SOD = superoxide dismutase; CAT = catalase; GSH-PX = glutathione peroxidase; T-AOC = total antioxidant capacity; MDA = malondialdehyde; CON = basal diet; and AOK = basal diet in which 3% of the mixed coarse material was replaced with AOK. Values with different superscripts (a, b) show significant differences at *p* ≤ 0.05, extremely significant differences at *p* ≤ 0.01, and trend values are considered when the probability is within the range of 0.05 < *p* < 0.10. Dashed lines serve as demarcation lines between different enzyme activities or proteins.

The effect of AOK on the activity of enzymes related to lipid metabolism and anti-oxidase activity in SADT of cashmere goats is shown in Figure 1. The activities of HSL (*p* = 0.027), ELOVL2 (*p* = 0.01), ELOVL5 (*p* = 0.046), CD36 (*p* = 0.013), SLC27A4 (*p* = 0.021), and FABP4 (*p* = 0.04) in the AOK group were significantly higher than those in the CON group, but the activities of FAS (*p* = 0.002), LPL (*p* = 0.048), and SCD (*p* = 0.026) were significantly lower than those in the CON group. There was no significant difference in MDH (*p* = 0.446) or ACC (*p* = 0.766) activities between the two groups (Figure 1a). The SOD (*p* = 0.032), CAT (*p* = 0.010), and GSH-PX (*p* = 0.029) activities and T-AOC (*p* = 0.002) in the AOK group were significantly higher than those in the CON group. No significant difference was observed in MDA (*p* = 0.469) content between the two groups (Figure 1b).

### 3.4. Statistics and Alignment Analysis of Sequencing

#### 3.4.1. Differentially Expressed Genes (DEGs)

**Figure 2:**
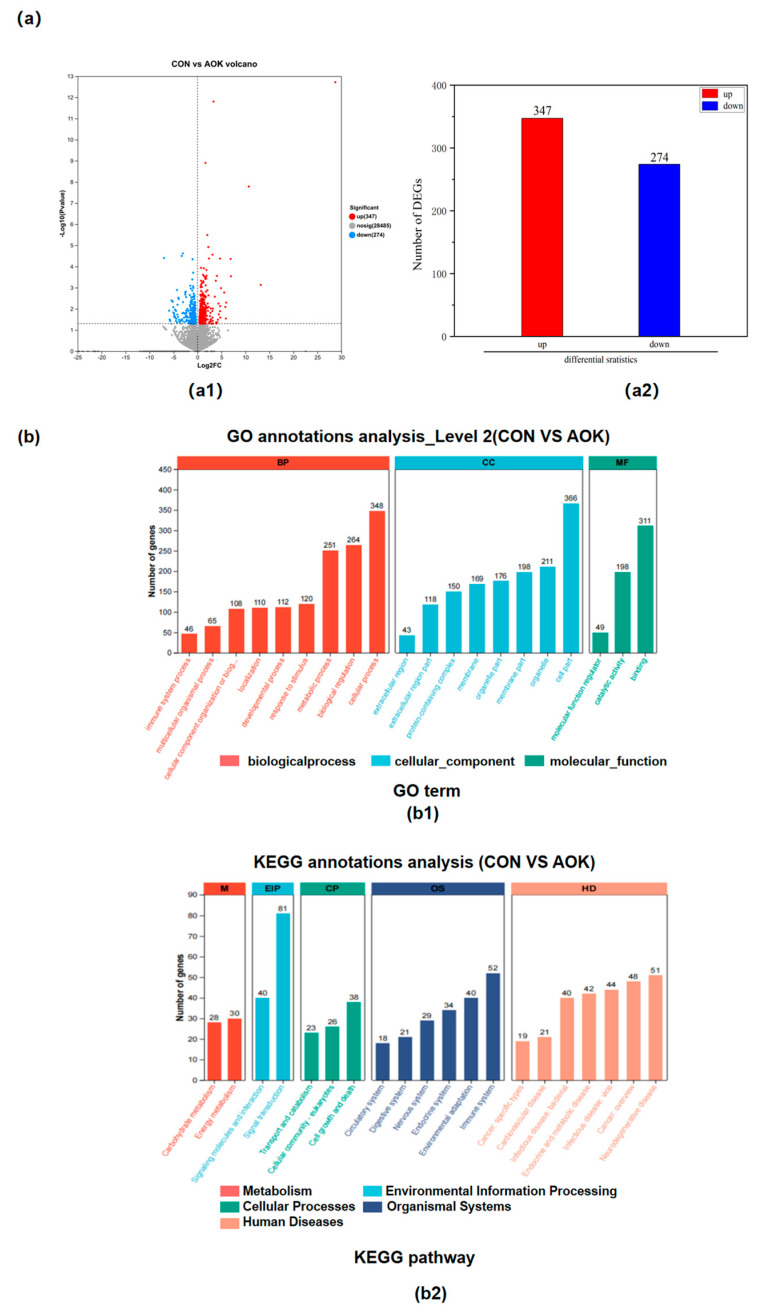
(**a**) Effect of AOK on DEGs in the SADT of cashmere goats; CON = basal diet; AOK = basal diet in which 3% of the mixed coarse material was replaced with AOK: (**a1**) volcano plot showing differences in expression levels; Vertical dashed lines represent the fold change threshold, while horizontal dashed lines indicate the significance (*p*-value) threshold. (**a2**) number of DEGs. (**b**) GO and KEGG annotation analysis results of DEGs: (**b1**) GO annotation results of DEGs in SADT; (**b2**) KEGG annotation results of DEGs in SADT.

The effect of AOK on DEGs in the SADT of cashmere goats is shown in Figure 2a. Following alignment with the goat reference genome, 3567 genes were identified in the SADT. Differential expression analysis between the CON and the AOK groups revealed 621 DEGs, comprising 347 significantly upregulated and 274 significantly downregulated genes (Figure 2a).

#### 3.4.2. GO and KEGG Annotation Analysis

The results of DEG annotation using GO and KEGG annotation are shown in Figure 2b. GO annotation analysis identified 20 pathways in total, including 9 pathways in biological processes, 8 pathways in cellular components, and 3 pathways in molecular function (Figure 2(b1)). KEGG annotation analysis also identified 20 pathways in total, including 7 pathways in human diseases, 6 pathways in organismal systems, 3 pathways in cellular processes, and 2 pathways in metabolism and environmental information processing (Figure 2(b2)).

#### 3.4.3. Lipid-Metabolism-Related Enriched Pathways

The results of KEGG enrichment analysis for DEGs are shown in Table A3. A total of 18 lipid-metabolism-related pathways were identified, among which 6 pathways were significantly enriched. These included glucose uptake (*p* = 0.002), adenosine-monophosphate-activated protein kinase (AMPK) signaling (*p* = 0.010), p53 tumor suppressor (P53) signaling (*p* = 0.015), phosphatidylinositol 3-kinase-akt (PI3K-Akt) signaling (*p* = 0.025), insulin resistance (*p* = 0.048), and cholesterol metabolism (*p* = 0.049). One pathway that approached significant enrichment was the peroxisome-proliferator-activated receptor (PPAR) signaling pathway (*p* = 0.051). The genes that were significantly enriched in the AMPK signaling pathway included *LPL*, *SCD1*, *CPT1B*, and *GYS1*. Genes significantly enriched in the PI3K-Akt signaling pathway included *GYS1* and *ERBB4*. Genes significantly enriched in the insulin resistance signaling pathway included *CPT1B*, *ADCY2*, and *GYS1*. The genes significantly enriched in the cholesterol metabolism signaling pathway included *LPL*, and those showing a tendency toward significant enrichment in the PPAR signaling pathway included *ACSL4*, *CPT1B*, *SCD1* and *LPL*. Among these, only *ACSL4* was upregulated, while the other DEGs were downregulated.

#### 3.4.4. Validation of RNA-Seq Data by qRT-PCR

**Figure 3:**
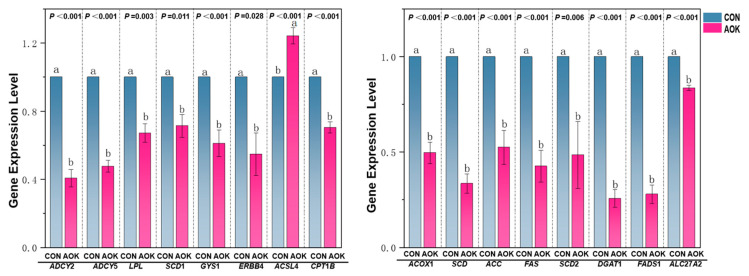
Effects of dietary AOK supplementation on the expression of genes related to SADT in cashmere goats. Abbreviations: *ADCY*2 = *adenylyl cyclase* 2; *ADCY*5 = *adenylyl cyclase* 5; *LPL* = *lipoprotein lipase*; *SCD*1 = *stearoyl-CoA desaturase*1; *GYS*1 = *glycogen synthase* 1; *ERBB*4 = *erythroblastic leukemia viral oncogene homolog* 4; *ACSL*4 = *acyl-coa synthetase long-chain family member* 4; *CPT*1*B* = *carnitine palmitoyltransferase* 1*b*; *ACOX*1 = *acyl-coa oxidase* 1; *SCD*2 = *stearoyl-CoA desaturase* 2; *DGAT*1 = *diacylglycerol acyltransferase* 1; *FADS*1 = *fatty acid desaturase* 1; *SLC*27*A*2 = *solute carrier family* 27 *member* 2; CON = basal diet; AOK = basal diet in which 3% of the mixed coarse material was replaced with AOK. Values with different superscripts (a, b) show significant differences at *p* ≤ 0.05, extremely significant differences at *p* ≤ 0.01, and trend values are considered when the probability is within the range of 0.05 < *p* < 0.10.

The effect of AOK on the gene expression of enzymes related to lipid metabolism in the SADT of cashmere goats is shown in Figure 3. The expression levels of the *ADCY*2, *ADCY*5, *LPL*, *SCD*1, *GYS*1, *ERBB*4, *ACOX*1, *SCD*, *ACC*, *FAS*, *SCD*2, *DGAT*1, *FADS*1, *SLC*27*A*2, and *CPT*1*B* genes in the SADT of the AOK group were significantly lower than those in the CON group (*p* < 0.05), whereas the expression level of the *ACSL*4 gene in the SADT of the AOK group was significantly higher than that in the CON group (*p* < 0.001). Despite some variations in the magnitude of fold-change for specific genes, the overall concordance in gene expression trends with the transcriptome sequencing data supports the validity and reliability of the RNA sequencing results.

## 4. Discussion

The FA composition of SADT is an important indicator affecting meat quality [25]. Excessive C16:0 can increase plasma lipoproteins [26], C18:0 may lead to a gamey taste in lamb [27], while C18:1n9 can reduce blood cholesterol and low-density lipoprotein levels [28]. In this experiment, the SFA content in the SADT of goats treated with AOK was significantly lower than that in CON group, whereas the MUFA content was significantly higher. Figure 1 shows that the FA composition changes in the SADT of cashmere goats were due to metabolic regulation rather than differences in dietary FAs. Dietary supplementation with AOK can promote the conversion of more SFAs to MUFAs in SADT, which is consistent with the findings of Meng et al. [29]. Guo et al. [30] reported similar results in a study where 2.5 g/kg plant nutrient additives were added to the diet of female lambs. However, the results of this experiment are inconsistent with those of Biligel et al. [31], possibly due to differences in dosage. N3-PUFAs have unique physiological functions and cannot be converted in the body, so they must be supplied through the diet [32]. The significant increase in n3-PUFA content within the SADT of AOK-supplemented goats suggests a direct regulatory effect of AOK on local lipid metabolism. This effect is likely attributed to the inhibition of lipid oxidation, a mechanism supported by evidence showing that plant extracts can preserve PUFAs by altering the activity of key metabolic enzymes [33]. Although Choi et al. [34] reported comparable findings for AOK, the specific pathways involved remain to be fully elucidated.

The hydrolysis of TG in SADT adipocytes produces glycerol and free fatty acids (FAs). The released glycerol can be exported to the liver for gluconeogenesis, while the FAs undergo β-oxidation for energy production. The content of C18:3n3 in the SADT of the AOK group was significantly higher than that in the CON group, which indicates that AOK influences both fat synthesis and decomposition in various ways.

AMPK can reduce lipid deposition by upregulating the expression of genes in the fat decomposition pathway such as *PPARα* and *CPT*1*B* by downregulating the expression of genes in the fat synthesis pathway, such as *SREBP*1 [35]. AOK may control the AMPK pathway by regulating these genes, thereby reducing TG synthesis in SADT and modulating FA content. Both SCD and FAS are generally considered rate-limiting enzymes in FA biosynthesis. SCD catalyzes the conversion of SFAs to PUFAs [36]. AOK can significantly downregulate the expression of *SCD*, *SCD*1, and *SCD*2, thereby activating the AMPK/PPARα pathway and increasing the PUFA content in SADT, which is consistent with the findings of Ntambi et al. [37]. FAS is also generally considered a rate-limiting enzyme in FA biosynthesis. In this experiment, intake of AOK reduced *FAS* expression, which may reduce FA de novo synthesis by activating the AMPK/AREBP1c pathway, which is consistent with the previous discovery that AOK can significantly reduce TG in the plasma of cashmere goats [21]. *DGAT*1 in SADT is a key candidate gene involved in TG synthesis. Inhibition of DGAT activity in adipose tissue can reduce TG accumulation in adipocytes and improve body fat distribution [38]. In this study, mRNA expression levels of genes related to fat synthesis and storage (*ACC*, *FAS*, and *DGAT*1) were significantly decreased, consistent with the findings of Guo et al. [39]. Lu et al. [40] reported that plant extracts could inhibit the AMPK-SREBP1c-ACC/FASN pathway, thereby suppressing lipid synthesis. LPL and HSL are enzymes that hydrolyze TG [41,42]. LPL hydrolyzes TG to provide FAs for the storage of SADT [43]. In the AOK group, LPL activity was significantly decreased, while HSL activity was significantly increased. Lim et al. [44] found that the expression of *LPL* was decreased in mice after ingesting Bupleurum extract, which was consistent with our results. Zhang et al. [45] found that adding sand onion polysaccharides to the diet of sheep could significantly increase the expression of *HSL* mRNA. These indicate that AOK can promote TG decomposition by regulating the activities of LPL and HSL, thereby creating favorable conditions for increased n3-PUFAs. Modulating the expression of TG synthesis or hydrolysis factors can block the AMPK pathway, thereby reducing TG synthesis and increasing free FA content [46]. The expression levels of *SCD*, *SCD*1, *SCD*2, *DGAT*1, and *LPL* in SADT of the AOK group were downregulated, which is consistent with the findings of Seo et al. [47].

Both ELOVL2 and ELOVL5 function as key initiating and rate-limiting enzymes involved in PUFA synthesis [48]. The activities of ELOVL2 and ELOVL5 in the AOK group were significantly increased, suggesting that AOK can improve the conversion of C18:1n9 to n3-PUFAs in the SADT of cashmere goats, which is consistent with the results of Wang et al. [15]. CD36 can optimize the uptake of FAs by tissues [49], SLC27A4 can promote the uptake of n3-PUFAs [50], and FABP4 is mainly responsible for the transport of FAs on the cell membrane [51]. The results of this experiment showed that AOK significantly increased the levels of CD36, FABP4, and SLC27A4 in SADT, which is consistent with the findings of Dai et al. [52]. Comprehensive analysis indicates that AOK may increase the content of n3-PUFAs by reducing TG synthesis in SADT and enhancing FA transport capacity.

FA synthesis is closely related to glucose metabolism. Previous studies have found that dietary supplementation with AOK can reduce the glucose content in the plasma of cashmere goats [19]. When the blood glucose concentration is high, the body undergoes a process of “selective insulin resistance” [53], which can stimulate lipid deposition in adipocytes. Lipid metabolism can be regulated by modulating insulin secretion in the body, accelerating the decomposition of TG in adipocytes, and increasing FA content. The genes *ADCY*2 and *ADCY*5 are key regulatory factors in glucose and lipid metabolism, which can indirectly affect the aforementioned processes through insulin secretion. The expression levels of *ADCY*2 and *ADCY*5 in the SADT in the AOK group were decreased, which confirms with the previous finding that dietary supplementation with AOK can reduce blood glucose content in goats [19]. These results suggest that AOK may improve insulin resistance by reducing the expression levels of *ADCY*2 and *ADCY*5, thereby affecting TG decomposition in SADT, increasing FA content, and providing more precursor substances for the conversion of C18:1n9 to n3-PUFAs.

The PI3K-Akt pathway can reduce insulin secretion, increase glucose uptake and utilization, and enhance FA catabolism [54]. Activation of the PI3K-Akt pathway can inhibit lipid accumulation and promote fat metabolism [55]. *GYS1* is a key gene regulating glycogen synthase [56], and therefore, inhibiting its expression can reduce glycogen accumulation by activating the PI3K-Akt pathway. The upregulation of *ERBB4* can increase the binding and uptake of low-density lipoprotein by activating the PI3K-Akt pathway [57]. The expression levels of *GYS1* and *ERBB4* in the SADT of goats in the AOK group were significantly lower than those of the CON group, suggesting that AOK may enhance the aforementioned processes by activating the PI3K-Akt pathway. This finding is similar to the result reported by Luo et al. [58], who showed that kudzu activates the PI3K-Akt pathway in mice to improve insulin resistance.

FA synthesis and oxidation are important processes in lipid metabolism. According to the “double hit” hypothesis, there are two ways to reduce FA content: one is by increasing TG accumulation in adipose tissue leading to lipid metabolism disorders; the other is oxidative stress caused by excessive reactive oxygen species produced by lipid deposition, which causes lipid peroxidation. *ACSL*4 can positively regulate FA production in SADT. *CPT*1*B* is crucial for FA β-oxidation [59]. *ACOX*1 is a rate-limiting enzyme for n3-PUFA degradation. Decreased expression of *ACOX1* regulates the PPARα-mediated reduction in peroxisomal oxidation of n3-PUFAs [60]. The expression level of *ACSL*4 of the AOK group was significantly increased, while the expression levels of *CPT*1*B* and *ACOX*1 were significantly decreased and were enriched in the FA degradation pathway and adipocytokine signaling pathway. These results suggest that AOK may reduce the oxidation of n3-PUFAs in SADT. This finding is consistent with the mechanism found by Dai et al. [52], who showed that mulberry leaf water extract can improve liver glucose and lipid metabolism by activating the PI3K-Akt-PPARα-CPT1B pathway. Lu et al. reported that plant extracts could regulate the PPARα-CPT1/ACOX1 pathway to decrease FA oxidation [61]. *FADS1* can dehydrogenate C20:4n-3 to C20:5n-3. Reduction in *FADS*1 transcription levels is significantly associated with better accumulation of n3-PUFAs [62]. *SLC*27*A*2 converts free C20:4n-3/C20:5n-3 into acyl-CoA, which is closely related to the storage and activation of n3-PUFAs. The expression levels of *FADS*1 and *SLC*27*A*2 in the SADT of the AOK group were significantly lower than those in the CON group, suggesting that AOK enhances the storage ratio of C20:4n-3/C20:5n-3 in SADT by reducing the conversion and oxidation of n3-PUFAs, which is consistent with the results reported by Wu et al. [62].

**Figure 4:**
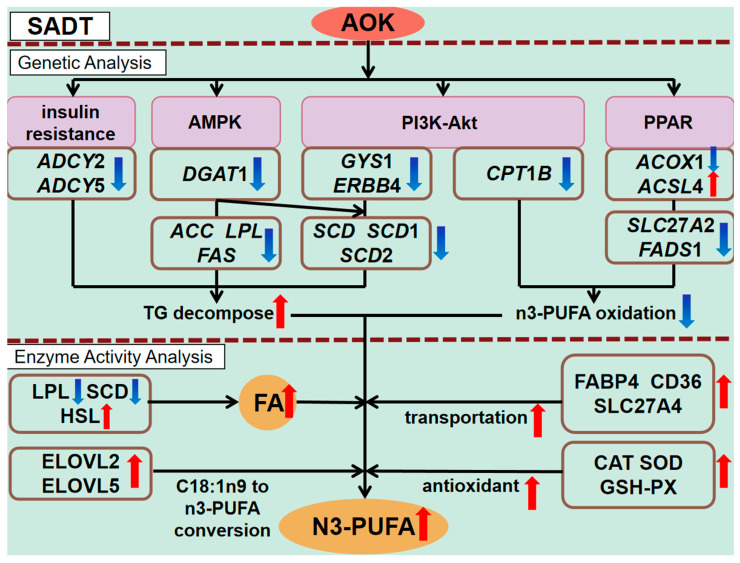
Effects of AOK intake on lipid-metabolism-related gene expression, enzyme activity, and antioxidant enzyme activity in the SADT of cashmere goats. Purple squares represent related signaling pathways. Red upward arrows indicate a significant increase, and blue downward arrows indicate a significant decrease.

The antioxidant enzyme activity in SADT is closely related to the content of n3-PUFAs. The double bonds of n3-PUFAs are highly susceptible to oxidative attack induced by reactive oxygen species (ROS). Enhancing CAT [63] and SOD can reduce degradation of n3-PUFAs by scavenging ROS, such as H_2_O_2_ and O_2_^−^ [64]. As a crucial enzymatic antioxidant, GSH-PX also prevents the peroxidation chain reaction of PUFAs by maintaining the redox balance within subcutaneous phagocytes [65]. In this study, the levels of SOD, CAT, and GSH-Px in the SADT of AOK-treated cashmere goats were significantly increased, which is consistent with the findings of Li et al. [66]. The AOK may increase free radical production by elevating PUFA content, thereby enhancing the likelihood of spontaneous lipid oxidation. Adipocytes may therefore enhance the activity of endogenous antioxidant enzymes to alleviate the resulting oxidative stress [67]. Additionally, AOK inherently contains a substantial amount of bioactive compounds with antioxidant functions, such as polysaccharides and flavonoids [6], which inhibit the formation of peroxides in PUFAs. MDA levels can be used as an indirect indicator of the extent of cellular injury. No significant difference in MDA levels was observed between the two groups, suggesting that AOK intake does not cause cellular damage and that the dosage is within a safe range. The effects of AOK supplementation on the expression of lipid-metabolism-related genes, enzyme activity, and antioxidant gene activity in SADT of cashmere goats are shown in Figure 4.

However, this study also has several limitations. First, the active components of AOK and their individual or combined effects were not identified in the present study. In addition, functional analysis of key metabolites was not performed due to the limited sample size. Therefore, these aspects will be further investigated in future studies to clarify the underlying mechanism.

## 5. Conclusions

In conclusion, AOK supplementation optimizes the FA profile in the SADT of cashmere goats by specifically increasing n3-PUFA content and lowering the n-6/n-3 ratio. These beneficial changes are mechanistically driven by enhanced synthesis and reduced β-oxidation of n3-PUFAs, coupled with promoted TG decomposition. Thus, AOK supplementation represents an effective strategy to enhance the nutritional quality of cashmere goat meat, mainly by increasing n3-PUFA accumulation in SADT.

## Figures and Tables

**Table 1:** Composition and nutrient levels of the CON and AOK diets (air-dry basis, %).

Ingredients	Early Phase		Middle Phase		Late Phase	
	CON	AOK	CON	AOK	CON	AOK
Alfalfa	25.00	22.00	15.00	14.10	12.50	11.75
Corn stalk	5.00	5.00	20.00	18.80	25.00	23.50
Oat	20.00	20.00	15.00	14.10	12.50	11.75
*Artemisia*	0.00	3.00	0.00	3.00	0.00	3.00
Corn	28.41	28.41	30.80	30.80	31.30	31.30
Soybean meal	11.70	11.70	9.50	9.50	8.00	8.00
DDGS ^1^	3.00	3.00	4.00	4.00	4.00	4.00
Linseed meal	4.80	4.80	3.50	3.50	4.50	4.50
Premix ^2^	0.50	0.50	0.50	0.50	0.50	0.50
NaCl	0.54	0.54	0.50	0.50	0.50	0.50
NaHCO_3_	0.35	0.35	0.80	0.80	0.80	0.80
MgO	0.30	0.30	0.00	0.00	0.00	0.00
CaHPO_4_	0.20	0.20	0.20	0.20	0.20	0.20
Limestone	0.20	0.20	0.20	0.20	0.20	0.20
Total	100.00	100.00	100.00	100.00	100.00	100.00
Nutrient levels ^3^						
DE (MJ/kg) ^4^	11.38	11.35	11.38	11.25	11.26	11.23
CP	88.71	88.58	88.39	88.35	88.42	88.41
EE	16.74	16.53	14.39	14.38	13.57	13.54
NDF	2.58	2.76	2.56	2.74	2.37	2.54
ADF	37.73	37.25	39.65	39.15	40.44	40.19
Ca	20.60	20.38	21.44	21.10	21.90	21.90
P	1.00	1.01	0.93	0.95	0.91	0.93

^1^ DDGS = distillers’ dried grains with solubles. ^2^ Premix provided the following per kilogram of diet: Fe 0.04 g, Cu 0.008 g, Zn 0.05 g, Mn 0.03 g, I 0.3 mg, Se 0.3 mg, Co 0.25 mg, VA 6000 IU, VD32500 IU, VE 12.5IU, VK 31.8 mg, VB 10.35 mg, VB 28.5 mg, VB 60.9 mg, nicotinic acid 22 mg, D-pantothenic acid 17 mg, VB 120.03 mg, biotin 0.14 mg, and folic acid 1.5 mg. ^3^ CP = crude protein, DE = digestible energy, EE = ether extract, NDF = neutral detergent fiber, ADF = acid detergent fiber, Ca = calcium, and P = phosphorus. ^4^ DE was calculated based on the ingredients of the diet and their digestible energy content, rather than on the actual dry matter intake. CON: basal diet; AOK: basal diet in which 3% of the mixed coarse material was replaced with AOK.

**Table 2:** FA intake (g/kg metabolic body weight).

FA ^1^	CON ^1^	AOK ^1^	SEM ^2^	*p*-Value
SFAs				
C10:0	0.007	0.009	0.002	0.659
C12:0	0.008	0.008	0.002	0.507
C13:0	0.002	0.002	0.001	0.987
C14:0	0.011	0.011	0.002	0.986
C15:0	0.006	0.006	0.001	0.974
C16:0	0.148	0.145	0.001	0.965
C17:0	0.007	0.007	0.000	0.868
C18:0	0.028	0.027	0.033	0.944
C20:0	0.012	0.012	0.001	0.978
C21:0	0.004	0.004	0.002	0.970
C22:0	0.012	0.012	0.001	0.974
MUFAs				
C14:1	0.003	0.003	0.031	0.975
C15:1	0.001	0.001	0.001	0.997
C16:1	0.006	0.006	0.069	0.970
C17:1	0.006	0.006	0.003	0.987
C18:1n9t	0.004	0.004	0.001	0.981
C18:1n9c	0.136	0.135	0.002	0.973
C20:1	0.007	0.007	0.030	0.910
C24:1	0.005	0.005	0.001	0.981
n3-PUFAs				
C18:3n3	0.133	0.128	0.003	0.987
C20:3n3	0.010	0.009	0.001	0.854
C20:5n3	0.007	0.007	0.001	0.876
C22:6n3	0.008	0.008	0.002	0.958
n6-PUFAs				
C18:2n6t	0.005	0.005	0.001	0.970
C18:2n6c	0.305	0.301	0.001	0.999
C18:3n6	0.004	0.004	0.001	0.864
C20:2n6	0.005	0.005	0.001	0.999
C20:3n6	0.003	0.002	0.002	0.977
C20:4n6	0.005	0.005	0.001	0.981
C22:2n6	0.004	0.004	0.002	0.992
Sum and Ratio				
SFAs	0.258	0.255	0.058	0.971
UFAs	0.660	0.649	0.149	0.959
MUFAs	0.172	0.171	0.039	0.975
PUFAs	0.488	0.478	0.110	0.953
n3-PUFAs	0.158	0.153	0.035	0.921
n6-PUFAs	0.330	0.326	0.075	0.969
n6/n3	0.129	0.137	0.030	0.869
U/S	0.106	0.112	0.025	0.871
P/S	0.078	0.082	0.018	0.867

^1^ SFAs = saturated fatty acids; UFAs = unsaturated fatty acids; MUFAs = monounsaturated fatty acids; PUFAs = polyunsaturated fatty acids; n3-PUFAs = n3-polyunsaturated fatty acids; n6-PUFAs = n6-polyunsaturated fatty acids; n6/n3 = n6-PUFAs/n3-PUFAs; U/S = UFAs/SFAs; P/S = PUFAs/SFAs. SFAs = C10:0 + C12:0 + C13:0 + C14:0 + C15:0 + C16:0 + C17:0 + C18:0 + C20:0 + C21:0 + C22:0; MUFAs = C14:1 + C15:1 + C16:1 + C17:1 + C18:1n9t + C18:1n9c + C20:1 + C22:1; n3-PUFAs = C18:3n3 + C20:3n3 + C20:5n3 + C22:6n3; n6-PUFAs = C18:2n6t + C18:2n6c + C18:3n6 + C20:2n6 + C20:3n6 + C20:4n6 + C22:2n6; UFAs = total FAs − SFAs; UFAs = MUFAs + PUFAs; PUFAs = n3-PUFAs + n6-PUFAs. n6/n3 = n6-PUFAs/n3-PUFAs; P/S = PUFAs/SFAs; U/S = UFAs/SFAs; CON = basal diet; AOK = basal diet in which 3% of the mixed coarse material was replaced with AOK. ^2^ SEM represents the standard error of the mean.

**Table 3:** Effect of AOK on FA composition of SADT in cashmere goats (%).

FA	CON	AOK	SEM	*p*-Value ^1^
SFAs				
C10:0	0.508 ^b^	1.298 ^a^	0.110	0.004
C12:0	0.502 ^b^	1.162 ^a^	0.030	0.026
C13:0	0.211 ^b^	0.475 ^a^	0.018	0.004
C14:0	4.260 ^a^	3.888 ^b^	0.011	0.030
C15:0	0.860 ^b^	1.813 ^a^	0.027	<0.0001
C16:0	29.882 ^a^	21.884 ^b^	0.503	0.049
C17:0	3.018 ^b^	3.984 ^a^	0.061	0.002
C18:0	20.671 ^a^	12.004 ^b^	1.001	0.023
C20:0	0.428 ^b^	1.116 ^a^	0.020	<0.0001
C21:0	1.085 ^a^	0.480 ^b^	0.039	<0.0001
C22:0	0.269 ^b^	0.910 ^a^	0.011	<0.0001
MUFAs				
C14:1	0.217 ^b^	0.636 ^a^	0.010	<0.0001
C15:1	0.138 ^b^	0.167 ^a^	0.005	0.001
C16:1	1.773 ^b^	4.471 ^a^	0.122	<0.0001
C17:1	1.269 ^b^	2.759 ^a^	0.149	0.002
C18:1n9t	0.168	0.212	0.013	0.237
C18:1n9c	26.913	28.226	0.965	0.654
C20:1	0.249 ^b^	0.690 ^a^	0.020	<0.0001
C24:1	0.145 ^b^	0.484 ^a^	0.010	<0.0001
n3-PUFAs				
C18:3n3	1.141 ^b^	2.218 ^a^	0.106	<0.0001
C20:3n3	0.147 ^b^	0.468 ^a^	0.011	<0.0001
C20:5n3	0.163 ^b^	0.532 ^a^	0.012	<0.0001
C22:6n3	0.181 ^b^	0.586 ^a^	0.010	<0.0001
n6-PUFAs				
C18:2n6t	0.155 ^b^	0.644 ^a^	0.034	<0.0001
C18:2n6c	3.647 ^b^	5.493 ^a^	0.076	<0.0001
C18:3n6	0.201 ^b^	0.501 ^a^	0.011	<0.0001
C20:2n6	0.180 ^b^	0.504 ^a^	0.010	<0.0001
C20:3n6	0.147 ^b^	0.468 ^a^	0.007	<0.0001
C20:4n6	0.401	0.453	0.018	0.063
C22:2n6	0.139 ^b^	0.461 ^a^	0.008	<0.0001
Sum and Ratio				
SFAs	61.872 ^a^	49.013 ^b^	2.953	0.008
UFAs	37.462 ^b^	49.286 ^a^	1.390	<0.0001
MUFAs	31.247 ^b^	37.644 ^a^	1.439	0.007
PUFAs	6.522 ^b^	12.314 ^a^	0.175	<0.0001
n3-PUFAs	1.632 ^b^	3.803 ^a^	0.130	<0.0001
n6-PUFAs	4.890 ^b^	8.511 ^a^	0.072	<0.0001
n6/n3	3.000 ^a^	2.266 ^b^	0.035	0.002
U/S	0.632 ^b^	1.013 ^a^	0.053	0.002
P/S	0.109 ^b^	0.252 ^a^	0.006	<0.0001

^1^ In the same row, values with different superscripts (a, b) show significant differences at *p* ≤ 0.05, extremely significant differences at *p* ≤ 0.01, and trend values are considered when the probability is within the range of 0.05 < *p* < 0.10. The same as below.

## Data Availability

The sequencing data are available from the National Center for Biotechnology Information under the Sequence Read Archive (SRA) with the BioProject No. SAMN55215649, SAMN55215650, SAMN5515651, SAMN5215652, SAMN55015653, and SAMN55115654.

## Abbreviations

The following abbreviations are used in this manuscript:

PUFAs	Polyunsaturated fatty acids
n3-PUFAs	n3-polyunsaturated fatty acids
n6-PUFAs	n6-polyunsaturated fatty acids
n6/n3	n6-PUFAs/n3-PUFAs
U/S	UFAs/SFAs
P/S	PUFAs/SFAs
SADT	Subcutaneous adipose tissue
SFAs	Saturated fatty acids
MUFAs	Monounsaturated fatty acids
UFAs	Unsaturated fatty acids
AOK	*Artemisia ordosica* Krasch.
DDGS	Distillers’ dried grains with solubles
FAS	Fatty acid synthase
FADS	Fatty acid desaturases
ACC	Acetyl-CoA carboxylase
LPL	Lipoprotein lipase
HSL	Hormone-sensitive lipase
SCD	Stearoyl-CoA desaturase
ELOVL2	Elongase of very-long-chain fatty acids 2
ELOVL5	Elongase of very-long-chain fatty acids 5
MDH	Malate dehydrogenase
CD36	Cluster of differentiation 36
SLC27A4	Solute carrier family 27 member 4
FABP4	Fatty acid binding protein 4
SOD	Superoxide dismutase
CAT	Catalase
GSH-PX	Glutathione peroxidase
T-AOC	Total antioxidant capacity
MDA	Malondialdehyde
SREBP1	Sterol regulatory element-binding protein 1
GYS1	Glycogen synthase 1
ACSL4	Acyl-CoA synthetase long-chain family member 4
CPT1B	Carnitine palmitoyltransferase 1B
ERBB4	Erythroblastic leukemia viral oncogene homolog 4
ADCY2	Adenylyl cyclase 2
ADCY5	Adenylyl cyclase 5
ACOX1	Acyl-CoA oxidase 1
SCD1	Stearoyl-CoA desaturase 1
SCD2	Stearoyl-CoA desaturase 2
DGAT1	Diacylglycerol acyltransferase 1
FADS1	Fatty acid desaturase 1
SLC27A2	Solute carrier family 27 member 2
AMPK	Adenosine-monophosphate-activated protein kinase
P53	p53 tumor suppressor
PI3K-Akt	Phosphatidylinositol 3-kinase-akt
PPAR	Peroxisome-proliferator-activated receptor
MAPK	Mitogen-activated protein kinase
TG	Triglyceride

## Appendix A

**Table A1:** FA composition of the experimental diet (% of total FAs).

FA ^1^	Early Phase		Middle Phase		Late Phase		AOK
	CON ^1^	AOK ^1^	CON	AOK	CON	AOK	
SFAs							
C10:0	0.76	0.91	0.83	0.97	0.82	0.96	9.78
C12:0	0.9	0.91	0.94	0.94	0.93	0.93	1.85
C13:0	0.25	0.25	0.25	0.25	0.25	0.25	0.75
C14:0	1.16	1.18	1.17	1.18	1.16	1.16	2.64
C15:0	0.61	0.62	0.62	0.61	0.61	0.61	1.12
C16:0	16.35	16.12	16.26	16.13	15.81	15.72	13.08
C17:0	0.78	0.78	0.8	0.8	0.8	0.8	1.40
C18:0	3.06	3.04	3.05	3.02	3.01	2.99	3.28
C20:0	1.29	1.32	1.3	1.33	1.29	1.32	3.87
C21:0	0.39	0.39	0.4	0.4	0.39	0.39	1.11
C22:0	1.29	1.32	1.3	1.33	1.29	1.32	3.87
MUFAs							
C14:1	0.33	0.34	0.36	0.37	0.36	0.37	1.18
C15:1	0.16	0.15	0.13	0.12	0.12	0.11	0.12
C16:1	0.65	0.66	0.7	0.7	0.7	0.7	1.22
C17:1	0.58	0.59	0.62	0.62	0.62	0.62	1.02
C18:1n9t	0.47	0.49	0.46	0.47	0.44	0.45	1.09
C18:1n9c	13.51	13.66	15.39	15.38	15.78	15.76	7.33
C20:1	0.71	0.72	0.75	0.75	0.76	0.76	1.04
C22:1	0.4	0.39	0.4	0.38	0.42	0.4	0.00
n3-PUFAs							
C18:3n3	17.16	16.57	13.07	12.85	13.16	13	11.77
C20:3n3	1.05	1.05	1.05	1.05	1.04	1.04	1.70
C20:5n3	0.73	0.74	0.77	0.77	0.77	0.77	1.32
C22:6n3	0.76	0.78	0.91	0.92	0.95	0.96	2.45
n6-PUFAs							
C18:2n6t	0.55	0.56	0.5	0.51	0.48	0.49	1.08
C18:2n6c	31.68	31.75	34.06	34.06	34.18	34.18	22.23
C18:3n6	0.49	0.49	0.38	0.38	0.34	0.35	1.07
C20:2n6	0.54	0.55	0.55	0.55	0.55	0.55	1.38
C20:3n6	0.35	0.34	0.26	0.24	0.23	0.22	0.00
C20:4n6	0.54	0.54	0.49	0.5	0.46	0.47	1.23
C22:2n6	0.52	0.5	0.4	0.39	0.37	0.35	0.00
Sum and Ratio							
SFAs	28.3	28.3	28.3	28.34	27.67	27.76	42.75
UFAs	71.73	71.41	71.83	71.59	72.3	72.13	57.24
MUFAs	17.35	17.54	19.37	19.36	19.77	19.75	13.00
PUFAs	54.38	53.88	52.46	52.23	52.53	52.37	44.23
n3-PUFAs	19.7	19.14	15.81	15.6	15.92	15.77	17.24
n6-PUFAs	34.68	34.74	36.65	36.63	36.6	36.6	26.99
n6/n3	1.76	1.82	2.32	2.35	2.3	2.32	1.57
U/S	2.53	2.52	2.54	2.53	2.61	2.6	1.34
P/S	1.92	1.9	1.85	1.84	1.9	1.89	1.03

^1^ SFAs = saturated fatty acids; UFAs = unsaturated fatty acids; MUFAs = monounsaturated fatty acids; PUFAs = polyunsaturated fatty acids; n3-PUFAs = n3-polyunsaturated fatty acids; n6-PUFAs = n6-polyunsaturated fatty acids; n6/n3 = n6-PUFAs/n3-PUFAs; U/S = UFAs/SFAs; P/S = PUFAs/SFAs. SFAs = C10:0 + C12:0 + C13:0 + C14:0 + C15:0 + C16:0 + C17:0 + C18:0 + C20:0 + C21:0 + C22:0; MUFAs = C14:1 + C15:1 + C16:1 + C17:1 + C18:1n9t + C18:1n9c + C20:1 + C22:1; n3-PUFAs = C18:3n3 + C20:3n3 + C20:5n3 + C22:6n3; n6-PUFAs = C18:2n6t + C18:2n6c + C18:3n6 + C20:2n6 + C20:3n6 + C20:4n6 + C22:2n6; UFAs = total FAs − SFAs; UFAs = MUFAs + PUFAs; PUFAs = n3-PUFAs + n6-PUFAs. n6/n3 = n6-PUFAs/n3-PUFAs; P/S = PUFAs/SFAs; U/S = UFAs/SFAs; CON = basal diet; AOK = basal diet in which 3% of the mixed coarse material was replaced with AOK.

**Table A2:** Primer pair sequences for quantitative real-time PCR.

Gene	Primer Pairs (5′-3′)	Annealing Temperature (°C)	Length (bp)
*CPT1B*	F:tgttcaacaccactcgcatc R:ctcgtagagccacagcttga	58	116
*SCD1*	F:cccagctgtcagagaaaagg R:gatgaagcacaacagcagga	60	115
*LPL*	F:tcaggcgtctttgcgggtat R:gcctgtttcattgcgtttg	60	194
*ACSL4*	F:tgctgggagggaatgtccgtat R:accgccttcctgccagtctt	60	152
*ADCY2*	F:tcttcctccacacacacaccgct R:ggtggtataactcctcgttct	63	314
*ADCY5*	F:tgtctggtctaacgatgtc R:cgatactgtgctccttga	53	164
*GYS1*	F:gccgacagggtcaaggtaa R:cggagaggttggtggagat	60	190
*ERBB4*	F:ccggaattccggtttgctgtgttgttcggc R:ccggggtaccccggactgtcatttgggcttggta	57	1018
*ACOX1*	F:gagtgagctgcctgagcttc R:ttgtccaggacgtgaaagc	59	62
*SCD*	F:cccagctgtcagagaaag R:gatgaagcacacacagcagga	60	115
*ACC*	F:acccaacccagaaaggtcagt R:tcccacggattcctccta	60	125
*FAS*	F:gcaaccaggaggagaccgt R:ctgagggcaatggggatgg	60	300
*SCD2*	F:gttcagtgggcacctcaact R:tactcaatgccgtgcttgg	57	106
*DGAT1*	F:acgtcccggtaggaaaacag R:cgtggccttcctcctagagt	60	100
*FADS1*	F:atgcaacgtccacacaagtcag R:gggagccactttgtgtgttaat	56	115
*SLC27A2*	F:aaggccccgctttcttaag R:tcggtgtttaaaagttccagtg	56	61
*β-actin*	F:actgggacgacatggagaaga R:gcgtacagggacagcacag	60	199
*B2M*	F:ggtgctgcttagaggtctcg R:acgctgagttcactcccaac	59	109
*YWHAZ*	F:tgtaggagcccgtaggtcatct R:ttctctctgtattctcgagccatct	59	102

**Table A3:** Enrichment of KEGG pathways related to lipid metabolism in SADT.

Pathway Id	Description	DEGs	Background Number	*p*-Value	Upregulated Gene	Downregulated Gene
map00010	Glycolysis/ Gluconeogenesis	8	95	0.002	*-*	*TPI1*; *MYH1520*; *ALDH3A1*; *GAPDH*; *DLD*; *LOC108634096*; *PGM1*; *LOC106503669*
ma04152	AMPK signaling pathway	6	150	0.010	*-*	*LPL*; *IGF1*; *SCD1*; *CPT1B*; *PPP2R3C*; *GYS1*;
map04115	p53 signaling pathway	8	122	0.015	*LOC102176530*; *LOC108634455*; *LOC108634455*; *SERPINB5*; *PERP*;	*IGF1*; *LOC102176417*; *LOC102173997*
map04151	PI3K-Akt signaling pathway	19	512	0.025	*LOC106503653*; *ITGA2B*; *SYK*; *LOC108638107*; *CSF1*; *COMP*; *PKN3*; *ITGB3*; *COL9A3*; *SPP1*;	*GYS1*; *FGF13*; *IGF1*; *CDC37*; *THBS4*; *FGFR2*; *LOC108638200*; *PPP2R3C*; *ERBB4*;
map04931	Insulin resistance	3	125	0.048	*-*	*CPT1BADCY2*; *GYS1*;
map04979	Cholesterol metabolism	4	57	0.049	*APOE*;	*VDAC3*; *LPL*; *VDAC1*
map03320	PPAR signaling pathway	5	95	0.051	*ACSL4*;	*CPT1B*; *SCD1*; *LPL*; *DBI*
map00030	Pentose phosphate pathway	2	43	0.125	*PGLS*;	*PGM1*;
map04010	MAPK signaling pathway	15	123	0.157	*CSF1*; *DUSP2*; *RASGRF1*; *MAPK8IP1*; *CDC25B*; *MAPK11*	*FGF13*; *LOC108638200*; *IGF1*; *FGFR2*; *MAP2K6*; *ERBB4*; *DUSP10*; *ECSIT*; *CACNB4*
map00071	Fatty acid degradation	2	56	0.394	*ACSL4*	*CPT1B*;
map00061	Fatty acid biosynthesis	1	21	0.402	*ACSL4*	*-*
map00590	Arachidonic acid metabolism	4	136	0.418	*LTC4S*; *LOC102187204*; *PTGIS*; *LOC102178311*	*-*
map04923	Regulation of lipolysis in adipocytes	2	65	0.469	*-*	*ADCY5*; *ADCY2*
map01040	Biosynthesis of unsaturated fatty acids	1	33	0.555	*-*	*SCD1*
map04920	Adipocytokine signaling pathway	2	79	0.573	*ACSL4*	*CPT1B*;
map00561	Glycerolipid metabolism	2	86	0.619	*-*	*AGK*; *LPL*
map04975	Fat digestion and absorption	1	64	0.792	*-*	*GOT2*
map04910	Insulin signaling pathway	3	189	0.839	*PRKAR1B*;	*PHKG1*; *GYS1*
